# Expression of Concern: MicroRNA-140-5p inhibits cell proliferation, migration and promotes cell apoptosis in gastric cancer through the negative regulation of THY1-mediated Notch signaling

**DOI:** 10.1042/BSR-2018-1434_EOC

**Published:** 2022-10-26

**Keywords:** Gastric cancer, microRNA-140-5p, Migration, Notch signaling pathway, Proliferation, THY1

The Editorial Office has been made aware of potential issues surrounding the scientific validity of this paper, hence has issued an expression of concern to notify readers whilst the Editorial Office investigates. It is noted that two similar regions exist between the miR-140-5p inhibitor panel and the miR-140-5p inhibitor+si-Notch1 panel of Figure 7B, as well as the miR-140-5p inhibitor panel and the si-NC panel of Figure S3. Concerns also exist regarding the western blot images in Figures 2, 3, 5, 9, S1 and S5.

